# Supplementary material for: Synthesis and Biological Activity of Novel α-Conotoxins Derived from Endemic Polynesian Cone Snails
Source: Mar Drugs. 2023 Jun 9;21(6):356. doi: 10.3390/md21060356 (PMC10301793; doi:10.3390/md21060356)
Supplement: Supplementary file 1 [file marinedrugs-21-00356-s001.zip › marinedrugs-2429628-supplementary.pdf]

Supporting Information

Synthesis and Biological Activity of Novel alpha-conotoxins derived from Endemic Polynesian Cone Snails

Yazid Mohamed Souf [1], Gonxhe Lokaj [2], Veeresh Kuruva [3], Yakop Saed [3], Delphine Raviglione [1], Ashraf Brik [3], Annette Nicke[2], Nicolas Inguimbert [1]* and Sébastien Dutertre [4]*

[1] CRIOBE, USR CNRS-EPHE-UPVD 3278, Université de Perpignan Via Domitia. 58 avenue Paul Alduy 66860 Perpignan, FRANCE.
[2] Walther Straub Institute of Pharmacology and Toxicology, Faculty of Medicine, LMU Munich, Nußbaumstraße 26, 80336 Munich, Germany;
[3] Schulich Faculty of Chemistry, Technion-Israel Institute of Technology, Haifa 3200008, Israel
[4] IBMM, Université Montpellier, CNRS, ENSCM, 34093 Montpellier, France
* Correspondence: sebastien.dutertre@umontpellier.fr (SD) or nicolas.inguimbert@univ-perp.fr (NI);

Table of contents

General methods 1
Linear precursors 2
Table S1 : Amino acid sequence of linear conotoxin peptides synthesized 2
One pot regioselective approach 3
Figure S1: LC-MS, One pot regioselective oxidation of GaIA 3
Figure S2: LC-MS analysis One pot regioselective oxidation of AdIA 4
Figure S3: Temperature dependent HPLC; analyses of AdIA cis/trans conformations. 5
Figure S4 UHPLC-HRMS analysis synthetic folded GaIA 5
Figure S5 UHPLC-HRMS analysis of synthetic globular AdIA 6
Figure S6 : UHPLC-HRMS analysis of synthetic ribbon and globular AdIA 6
Oxidative folding 7
Figure S7: Various oxidative folding conditions tested on GaIA, ... 7
Figure S8: Various oxidative folding conditions tested on AdIA, ... 8
Figure S9: Comparison of oxidative folding and regioselective folding on AdIA 8

General methods

SPPS was carried by using an automated peptide synthesizer liberty Blue CEM (Charlotte, NC, USA) or CS336X (CSBIO, CA, USA). All the chemicals are analytical grade. N,N-dimethylformamide (DMF), dichloromethane (DCM), trifluoroacetic acid (TFA) and N,N-Diisopropylethylamine (DIEA) were purchased from Biolab (Jerusalem, ISL) or Honeywell (Charlotte, NC, USA) . triisopropylsilane (TIS), dithiothreitol (DTT), sodium dithiocarbamate (DTC), disulfiram (DSF), were purchased from Alfa Aesar (Haverhill, MA, USA)

Rink amide resins were purchased from CreoSalus (Louisville, KY, USA) or Sigma Aldrich (Saint-Louis, MO, USA). Fmoc protected amino acids were purchased from GL Biochem (Shanghai, CN), Iris Biotech (Marktredwitz, GE) or Sigma Aldrich and activating reagents, HCTU, OxymaPure were purchased from Luxembourg Bio Technologies (Ness Ziona, ILS) and Iris Biotech (Marktredwitz, GE).

Analytical HPLC was performed on a Thermo instrument (Dionex Ultimate 3000) using analytical Xbridge (Waters, BEH300 C4, 3.5 µm, 4.6 × 150 mm) columns at flow rate of 1.2 ml/min coupled to uv detectors at 214 nm.

LC-MS system consists of Thermo Fisher Scientific LC-MS device, Accela HPLC coupled to an QFleet fitted with an electrospray ionization source and an ion-trap analyzer. All analyzes were performed using a Kinetex 2.6 µm C18 column (150 x 3.00 mm) from Phenomenex Inc. (Torrance, CA, USA) in linear gradient mode from 2% to 60% over 30 min with a flow rate of 0.5 ml/ min (solvent A, water+0.1% FA; solvent B, acetonitrile+0.1% FA).

UHPLC/ HRMS system consists of a Vanquish UHPLC (Thermo Fisher Scientific, MA, USA) coupled to a QTOF Maxis II mass spectrometer (Brucker Daltonics, MA, USA), source electrospray ionization mode, ESI +. All analyzes were performed using a bioZen™ 2.6 µm Peptide XB-C18 column (150 x 2.1 mm, 100A) (Phenomenex, CA, USA) in linear gradient mode from 2% to 50% over 50 min with a flow rate of 0.3 ml/ min (solvent A, water+0.1% FA; solvent B, acetonitrile+0.1% FA)at 40°C. Acetonitrile, and formic acid RS for LC-MS (Carlo Erba, Val de Reuil, France), ultra-pure water from PURELAB Chorus 1 (ELGA Veolia, Lane End , UK).

All synthetic products were purified by HPLC and characterized by mass spectrometry using LCQ Fleet Ion Trap (Thermo Scientific). All calculated masses have been reported as an average isotope composition. Buffer A: 0.1% TFA in water; buffer B: 0.1% TFA in acetonitrile.

Linear precursors

Table S1 : Amino acid sequence of linear conotoxin peptides synthesized

Entry	Peptide	Sequence
1	GaIA*	H_2N-GRCCHPACGRKYNC-$CONH_2$
2	AdIA*	H_2N-GCCSTPPCAVLHC-$CONH_2$
3	GaIA (NBzl) *	H_2N-GRC(NBzl)CHPAC(NBzl)-GRKYNC-$CONH_2$
4	AdIA (NBzl) *	H_2N-GC(NBzl)CSTPPC(NBzl)AVLHC-$CONH_2$
5	GaIA (Acm) *	H_2N-GRC(Acm)CHPAC(Acm)-GRKYNC-$CONH_2$
6	AdIA (Acm) *	H_2N-GC(Acm)CSTPPC(Acm)AVLHC-$CONH_2$

* Trt-groups was remove after cleavage from resin

One pot regioselective approach

Bot approach have been done simultaneously (Nitrobenzyl, and Acm modified precursors), to observe the best result.

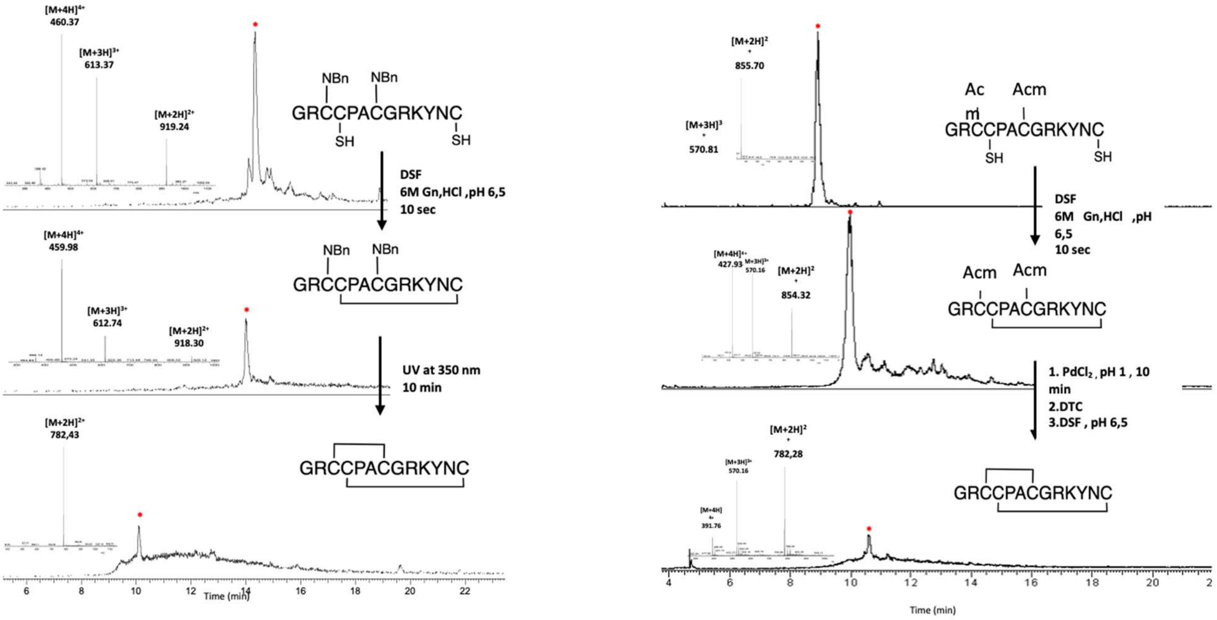

Figure S1: LC-MS, One pot regioselective oxidation of GalA

- **(A) Via NBzl:** The main peak observed corresponds to GalA modified with 2-nitrobenzyl at Cys I-III; After reaction with DSF, it leads to the formation of the first disulfide bond After UV radiation at 350nm for 10 minutes, GalA undergoes deprotection of NBn, followed by the formation of the second disulfide bond.
- **(B) Via Acm:** The main peak observed corresponds to GalA modified with Acm groups at Cys I-III (rt: 8.92 min). After Addition of DSF leads to the formation of the first disulfide bond. Palladium, DTC / DSF treatments, and conduct to the formation of second disulfide bonds was observed.

The difference in retention time for the final compound is explained by the treatment carried out after the end of the reaction for the two approaches. Simultaneous injection of the two samples results in a single identical peak.

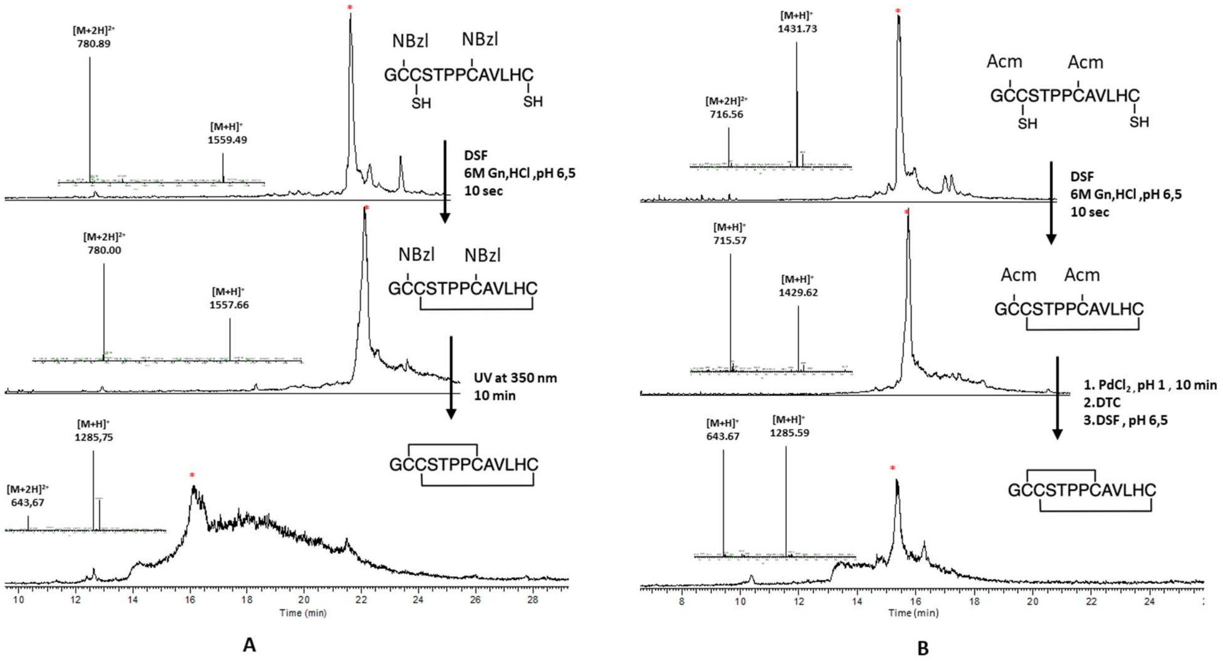

Figure S2: LC-MS analysis One pot regioselective oxidation of AdIA

- **(A) Via NBzl**: The main peak observed corresponds to AdIA modified with a 2-nitrobenzyl group at Cys I-III. Addition of DSF leads to the formation of the first disulfide bond. After UV radiation at 350nm for 10 minutes, AdIA undergoes deprotection of NBn, followed by the formation of the second disulfide bond.
- **(B) Via Acm**: The main peak observed corresponds to AdIA modified with Acm groups at Cys I-III. Addition of DSF leads to the formation of the first disulfide bond. After palladium treatment for 10 minutes, AdIA bearing two disulfide bonds was observed.

The difference in retention time for the final compound is explained by the treatment carried out after the end of the reaction for the two approaches. Simultaneous injection of the two samples results in a single identical peak.

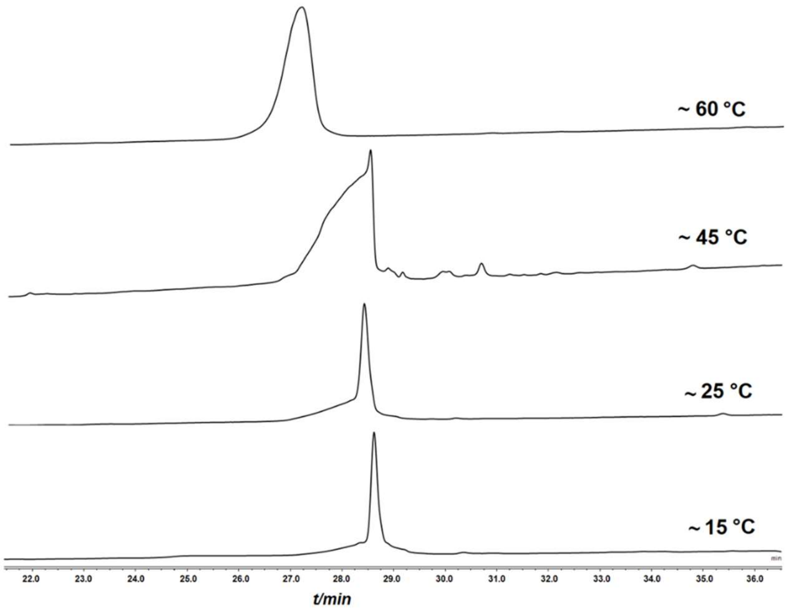

Figure S3: Temperature dependent HPLC; analyses of AdIA cis/trans conformations.

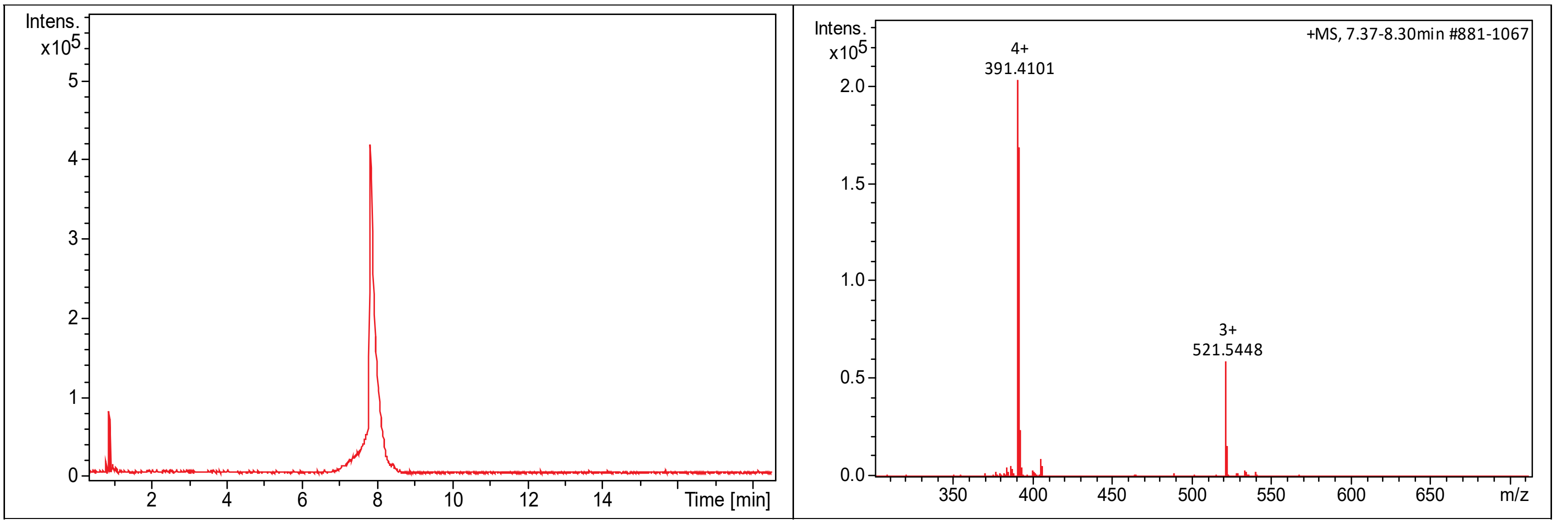

Figure S4 UHPLC-HRMS analysis synthetic folded GaIA

Purity ~97%

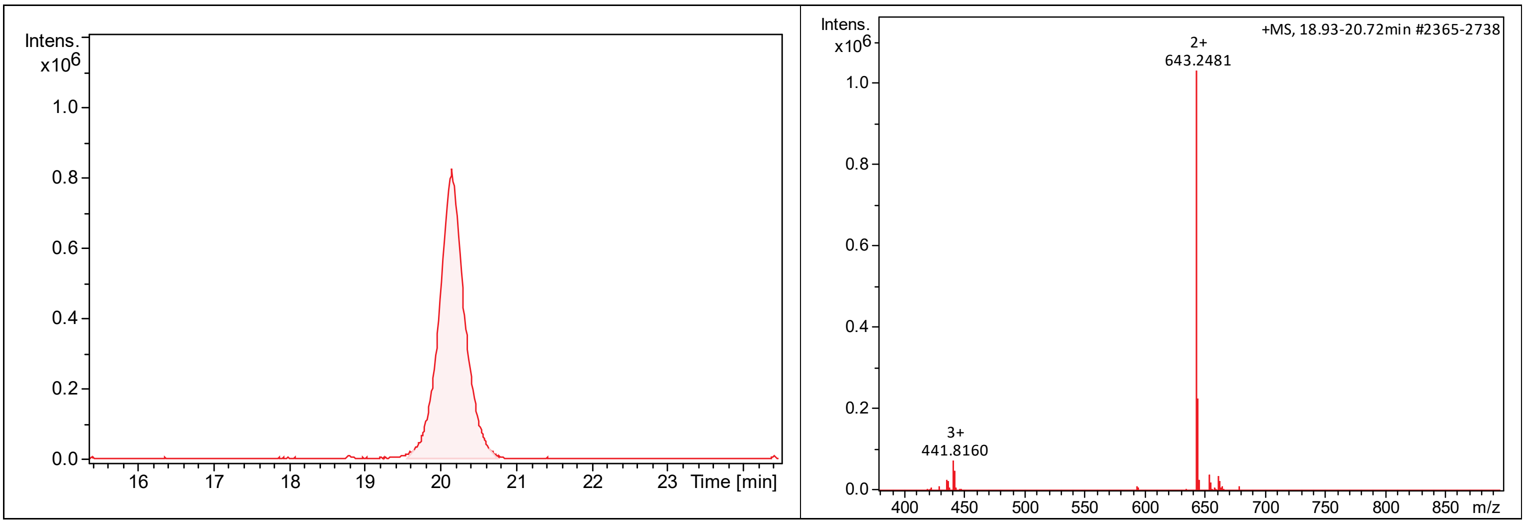

Figure S5 UHPLC-HRMS analysis of synthetic globular AdIA

Purity ~ 95%

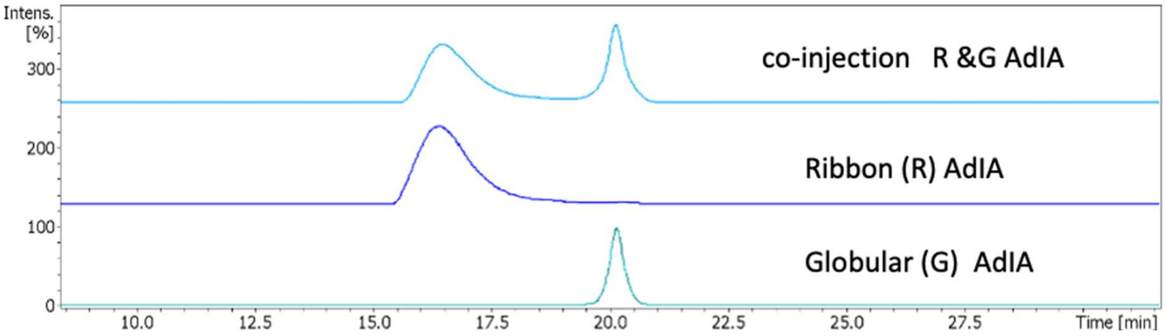

Figure S6 : UHPLC-HRMS analysis of synthetic ribbon and globular AdIA

Oxidative folding

For each condition, a 0.5 mg/ml linear fully deprotected precursor solution was prepared in the chosen buffer solution and left under agitation for 48 hours to lead to the native peptide. Once the reaction was complete, the reaction mixture was acidified to pH 3 with a 10% formic acid solution.

All analyzes were performed on Vanquish UHPLC (Thermo Fisher Scientific, MA, USA) coupled to a QTOF Maxis II mass spectrometer (Brucker Daltonics, MA, USA), source electrospray ionization mode, ESI +. using a bioZen™ 2.6 µm Peptide XB-C18 column (150 x 2.1 mm) (Phenomenex) in linear gradient mode from 2% to 98% with a flow rate of 0.4 ml/ min at 30°C (solvent A, water+0.1% formic acid; solvent B, acetonitrile+0.1% formic acid).

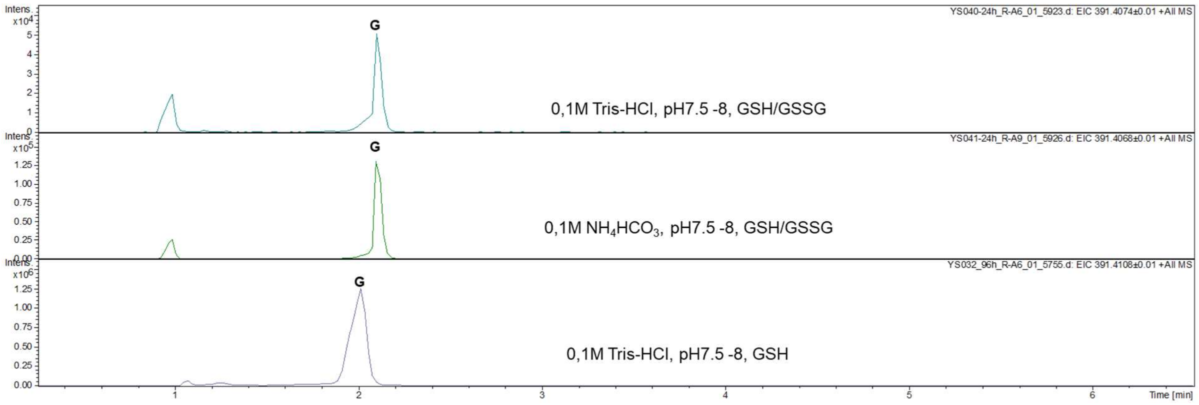

Figure S7: Various oxidative folding conditions tested on GalA

For GalA, most oxidative folding conditions lead to the correct isomer.

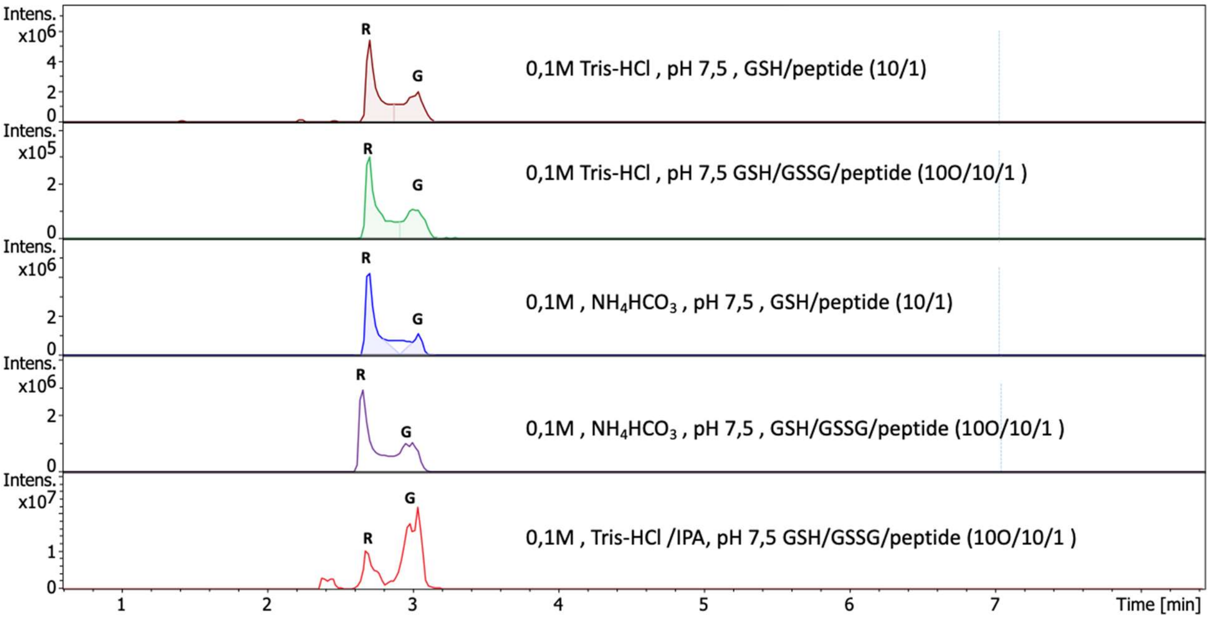

Figure S8: Various oxidative folding conditions tested on AdIA

G: globular isomer (Cys I-III, Cys II-IV, R: Ribbon isomer (Cys I-IV, Cys II-III). the peak splitting is caused by the two proline residues inducing cis-trans isomerization causing dynamic conformational exchange.

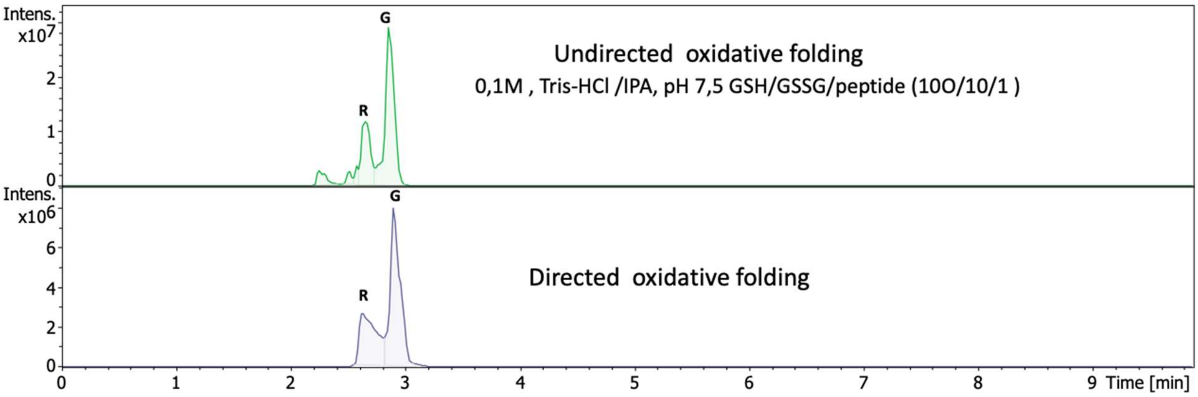

Figure S9: Comparison of oxidative folding and regioselective folding on AdIA

Analyzes were performed on Vanquish UHPLC (Thermo Fisher Scientific, MA, USA) coupled to a QTOF Maxis II mass spectrometer (Brucker Daltonics, MA, USA), source electrospray ionization mode, ESI +. using a bioZen™ 2.6 µm Peptide XB-C18 column (150 x 2.1 mm) (Phenomenex) in linear gradient mode from 2% to 98% with a flow rate of 0.4 ml/ min at **<u>60</u>°C** (solvent A, water+0.1% formic acid; solvent B, acetonitrile+0.1% formic acid).
